# Acupuncture therapy for gastric ulcer: A protocol for systematic review and meta-analysis

**DOI:** 10.1097/MD.0000000000027656

**Published:** 2021-10-29

**Authors:** Heran Wang, Hailin Jiang, Jinying Zhao, Xiaona Liu, Tie Li, Jiapeng Chai, Meng Meng, Ting Pan, Kaiyang Xu, Fuchun Wang

**Affiliations:** a School of Acupuncture-Moxibustion and Tuina, Changchun University of Chinese Medicine, Changchun; b Graduate School, Changchun University of Chinese Medicine, Changchun; c Department of Acupuncture, The Affiliated Hospital of Changchun University of Chinese Medicine, Changchun, China.

**Keywords:** acupuncture, gastric ulcer, protocol, systematic

## Abstract

**Background:**

Gastric ulcer (GU) is a clinically common disease of the digestive system that adversely affects patients’ quality of life and work ability. Although some articles have reported that acupuncture can improve the clinical symptoms of GU, the efficacy of acupuncture has not been scientifically or methodically evaluated. This study aimed to evaluate the effectiveness and safety of acupuncture for the treatment of patients with gastric ulcers.

**Methods:**

The following electronic databases will be searched from the respective dates of database inception to March 23, 2021: The Cochrane Library, Web of Science, EMBASE, MEDLINE, China National Knowledge Infrastructure, Chinese Biomedical Literature Database, Wanfang database, the Chinese Scientific Journal Database, and other sources. Randomized controlled trials comparing acupuncture with other interventions or sham acupuncture were included. Two independent researchers will perform article retrieval, duplication removal, screening, quality evaluation, and data analyses by Review Manager (V.5.3.5). Meta-analyzes, subgroup analysis, and/or descriptive analyses will be performed based on the included data conditions.

**Results:**

The protocol of this study systematically assessed the effectiveness and safety of acupuncture for gastric ulcer patients. The primary outcome was the effective rate, and the secondary outcomes included negative conversing rate of Helicobacter pylori infection, untoward effect, recurrence rate, quality of life, and symptom scores.

**Conclusion:**

This study provides evidence of whether acupuncture is an effective and safe intervention for gastric ulcers.

**PROSPERO registration number:**

CRD42021251067

## 1 Introduction

Gastric ulcer (GU), a common digestive disease, has a high incidence and seriously endangers human health, and is a chronic disease that affects up to 10% of the world's population.^[1]^ The estimated prevalence of peptic ulcer disease in the general population is 5% to 10%.^[2]^ A peptic ulcer occurs in areas exposed to acid and pepsin and is defined as a break in the mucosa lining the stomach or proximal intestine extending through the muscularis mucosae. Gastric ulcer is a localized tissue damage of the gastric mucosa caused by an increase in gastric acid and pepsin levels in the human body.^[3]^ Classic peptic ulcer disease is a chronic recurring disease that represents defective wound healing, and its complications include upper gastrointestinal bleeding, perforation, and, rarely, gastric outlet obstruction.^[4]^ A gastric ulcer in the body of the stomach signifies the presence of corpus gastritis; the more proximal the ulcer, the more extensive and severe the gastritis. Gastric cancer is also strongly associated with pangastritis. The incidence of gastric cancer increases with the extent and severity of gastritis.^[5,6]^ The most frequent causes of peptic ulcer disease are Helicobacter pylori infection and the use of non-steroidal anti-inflammatory drugs (NSAIDs), including aspirin. NSAIDs are among the most widely used drugs worldwide and are known to substantially increase the risk of upper gastrointestinal complications.^[7]^ Some scholars have pointed out that in patients with gastric ulcers, the positive rate of Helicobacter pylori is 15% for those whose ulcer is located in the stomach.^[8]^ The positive rates of HP in patients with ulcers located in the antrum and horn of the stomach were 37% and 44%, respectively; males and females had significant differences in the positive rates of HP at each ulcer site, with the former having a higher positive rate.^[9]^ According to relevant research reports, 23% of recurrent ulcers, 33% of complicated ulcers, and 30% of gastric cancers could be avoided if all patients received eradication therapy within 30 days of their initial peptic ulcer diagnosis.^[10]^ Many chemically synthesized drugs can be used to cure and control gastric ulcers and associated issues, but none are specific for stress ulcers; moreover, they have many adverse effects that can cause more complications. Therefore, there is an urgent need to find alternative therapies that are safer and more effective for treating stress ulcers.^[11]^

TCM, which includes acupuncture and moxibustion, Chinese traumatology, and Chinese herbal products, has been integrated as an important part of healthcare in China. It has been used to treat various diseases.^[12]^ Acupuncture, a complementary and alternative therapy, has been used in China for thousands of years and has become increasingly popular in Western countries because of its significant effect and few side effects.^[13]^ Recently, according to the previous studies, it has been proved that acupuncture at acupoints had a good effect on GU. Acupuncture has attracted much attention owing to the extensive use of TCM. Both basic research and clinical practice have proved that acupuncture has an obvious effect on gastric ulcers, but the therapeutic mechanism has not been very clear yet.^[14]^ The current literature maintains that acupuncture is effective at decreasing gastric ulcers in postoperative patients. However, evidence for the effectiveness improvement of postoperative recovery and safety for patients with gastric ulcers is still inconclusive. Therefore, this study aimed to systematically and comprehensively search literature records. This study will address a new aspect related to published studies to explore the effectiveness and safety of acupuncture for gastric ulcer. The results will provide the latest evidence of acupuncture for gastric ulcer in both clinical practice and further research in the field.

## 2 Methods

### 2.1 Design and registration of the review

This systematic review was registered in the PROSPERO network (registration number: CRD42021251067).This is the website https://www.crd.york.ac.uk/PROSPERO/. We followed the Preferred Reporting Items for Systematic Reviews and Meta-analysis Protocol^[15]^ to accomplish the systematic review protocol. This study was conducted for the secondary collection and analysis of original RCT data; therefore, informed consent or ethical approval was not required.

### 2.2 Inclusion criteria for study selection

#### 2.2.1 Type of study

All randomized controlled trials (RCTs) on the application of acupuncture in the treatment of patients with gastric ulcers will be included with no language limitation. However, animal studies, case reports, case series, commentaries, reviews, non-controlled trials, and other studies that were repeatedly published or did not have access to complete data non-RCTs will be excluded. RCTs and blinded studies will be included. Published clinical trials that reported the efficacy and safety of acupuncture for gastric ulcers will be included.

#### 2.2.2 Types of participants

Patients who were diagnosed with gastric ulcer will be included, without limits on gender, race, nationality, and medical units.

#### 2.2.3 Types of interventions and comparisons

Interventions can be any type of acupuncture; multiple control interventions will be included: no treatment, placebo, and other interventions (e.g., cupping therapy, drugs, physical interventions, moxibustion). If the interventions and comparisons both contain acupuncture, the study will be excluded. Interventions of acupuncture combined with other therapies will be included only if these combinations are compared to other therapies.

#### 2.2.4 Types of outcome measures

The primary outcome is the effective rate, which is categorized as cure, markedly effective, effective, or ineffective according to clinical symptoms, degree of gastric mucosal lesion under gastroscopy, and pathological changes of gastric mucosa; the secondary outcomes include negative conversion rate of H. pylori infection, untoward effect, recurrence rate, quality of life, symptom scores(stomach ache, stomach distention, belching, acid reflux, etc.),and comparison of curative effect of pathological tissue.

### 2.3 Data sources

The main sources of information that will be obtained in this study include electronic resource databases, which will be searched from the respective dates of database inception to March 23, 2021. We plan to search eight English and Chinese electronic databases, including the Web of Science, Cochrane Library, PubMed, EMBASE, SinoMed, Wanfang, China Science and Technology Journal, and China National Knowledge Infrastructure databases, for potentially eligible studies. We will also search for dissertations, conference proceedings, and reference lists of relevant included studies.

### 2.4 Search strategy

**Figure 1:**
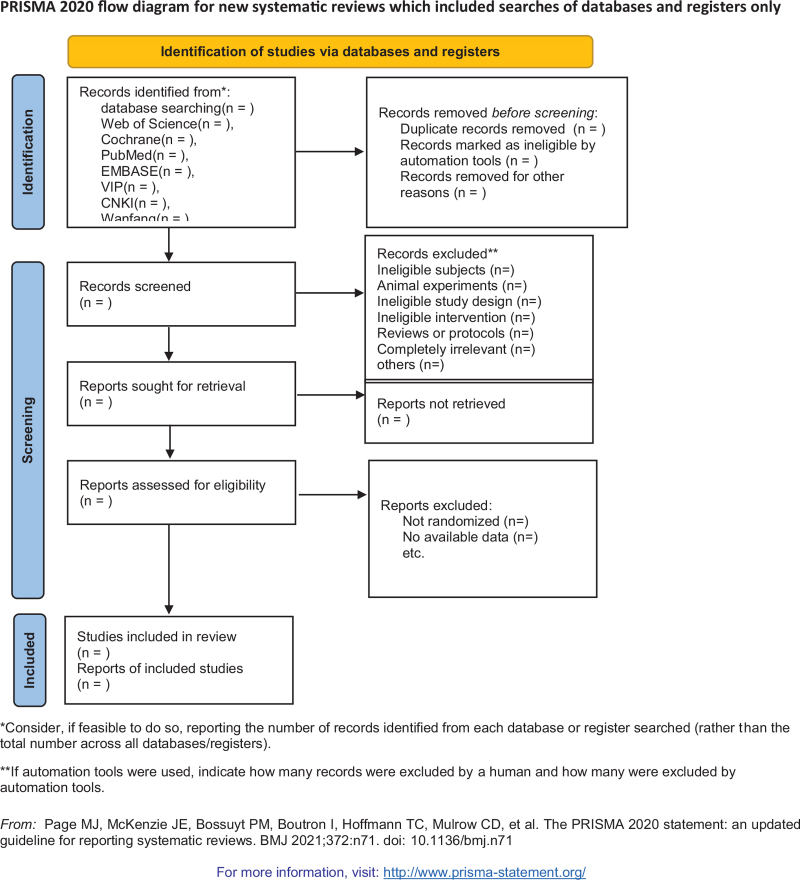
The PRISMA flow chart of selection process.

This strategy was created according to Cochrane Handbook guidelines. All published RCTs on this subject were included. The primary selection process is shown in the PRISMA flowchart (Fig. 1). The exemplary search strategy of WOS is listed in Table 1, and the search terms conform to the medical subject heading. According to the different retrieval modes, keywords may be combined with free words, and an appropriate search mode is performed. The search term will consist of 3 parts: disease, intervention method, and study type: (“gastric ulcer” or “gastrohelcosis” or “gastrohelcoma” or “GU” or “Peptic Ulcer”) and (“acupuncture” or “acupuncture and moxibustion therapy” or “acupuncture therapy” or “acupuncture point” or “acupuncture treatments” or “warm needling method” or “acupuncture and moxibustion” or “external application therapy” or “acupuncture point” or “Acupoints” or “electroacupuncture”) and (“randomized controlled trial” or “controlled clinical trial” or “random allocation” or “randomized” or “randomly” or “double-blind method” or “single-blind method” or “clinical trial”). Similar adaptive search strategies can be applied to other electronic databases. Language is restricted to English and Chinese. In addition, the following 3 trial registries will be searched for ongoing studies: Current Controlled Trials: www.controlled-trials.com; Clinical Trials: www.ClinicalTrials.gov; and Chinese Clinical Trial Registry: www.chictr.org.cn/index.aspx.

**Table 1:** Web of science search strategy.

Number	Terms
#1	Gastric ulcer
#2	Gastrohelcosis
#3	Gastrohelcoma
#4	GU
#5	Peptic Ulcer
#6	#1or#2-6
#7	Acupuncture
#8	Acupuncture and moxibustion therapy
#9	Acupuncture therapy
#10	Acupuncture point
#11	Acupuncture treatments
#12	Warm needling method
#13	Acupuncture and moxibustion
#14	External application therapy
#15	Acupuncture point
#16	Acupoints
#17	Electroacupuncture
#18	#7or#8-17
#19	Randomized controlled trial
#20	Controlled clinical trial
#21	Random allocation
#22	Randomized
#23	Randomly
#24	Double-blind method
#25	Single-blind method
#26	Clinical trial
#27	#19or#20–21

### 2.5 Data collection and analysis

#### 2.5.1 Selection of studies

Two authors will independently select clinical trials conforming to the inclusion criteria. After the articles were screened, disrelated, repetitive, nonstandard literature was excluded. We will also try to obtain the full text, and the obtained literature will be managed using EndNote software, V.X8 (United States). The selection process was presented in the PRISMA flow chart (http://www. Prismastatementorg/) (Fig. 1). If the full literature is unable to obtain or related data is incomplete, we will contact the corresponding author. Third-party experts will be consulted to determine selection divergence.

#### 2.5.2 Data extraction and management

Two independent authors collected data from the selected eligible articles entered into an Excel form. The extracted information included the reference ID, name of the first author, time of publication, country, participant characteristics, intervention, sample size, blinding, randomization, outcome measures, duration of follow-up, adverse effects, and other detailed information. If necessary, we will contact the corresponding authors of the trials as much as possible for further information.

#### 2.5.3 Assessment of the risk of bias and reporting of study quality

We independently evaluated the risk of bias to evaluate the quality of the studies using the Cochrane Collaboration's risk of bias assessment method. The following domains will be evaluated: random sequence generation, blindness of participants and staff, attrition bias, detection bias, selective result reporting, and other sources of bias. The risk of bias was assessed and classified according to 3 levels: low risk, unclear risk, and high risk. Any discrepancies were resolved through discussions and negotiations with the third author. When a consensus on risk assessment cannot be reached by discussion, the third reviewer will make the decision.

#### 2.5.4 Measures of treatment effect

Two authors independently and cross-checked the treatment effect using Review Manager 5.3.5, provided by the Cochrane Collaboration. Risk ratios with 95% confidence intervals were adopted for dichotomous data. Continuous data were presented as the mean difference or standard mean difference with a 95% confidence intervals. Other binary data were converted into risk ratio form for analysis.

#### 2.5.5 Management of missing data

We will try our best to ensure data integrity. If the necessary data in the literature may be lacking, we will contact the corresponding authors by email or other contacts. If the missing data are unavailable, an intent-to-treat analysis will be performed as much as possible (the analysis should include data from all participants in the initially randomly assigned group), and a sensitivity analysis will be performed to determine if the results are inconsistent.

#### 2.5.6 Assessment of heterogeneity

Cochrane Handbook for Systematic Reviews of Interventions^[16]^ The heterogeneity of studies will be evaluated by the X^2^ test will be used to detect statistical heterogeneity and the *I*^2^ statistic will be used to quantify inconsistency with Review Manager Software5.3.5. The following criteria were used: when the *I*^2^ test value was <50% and *P* value >1, we think there was no heterogeneity between these trials, and when the *I*^2^ test value was >50% and the *P* value was <1, there was significant heterogeneity between the included trials. A random-effects model was applied if heterogeneity was still important.

#### 2.5.7 Assessment of reporting bias

Funnel plots were created to assess reporting bias; once >10 trials were included, funnel plots were used to test for reporting bias. Dissymmetry funnel plots indicate a high risk of reporting bias, while symmetric funnel plots indicate low risk.

#### 2.5.8 Data synthesis

Review manager softwareV.5.3.5. software was used for all statistical analyses. We decided to use either a fixed-effects or random-effects model based on the heterogeneity levels of the included studies. If no substantial statistical heterogeneity is detected, the data synthesis will be processed using the fixed-effects model, and if substantial statistical heterogeneity is detected, the data synthesis will be performed using the random-effects model. If a significant level of heterogeneity was found, a descriptive analysis was performed.

#### 2.5.9 Subgroup analysis

Subgroup analysis was performed based on the findings of the data synthesis. Factors such as different types of control interventions and different outcomes will be considered, and subgroup analysis will be conducted relevant to these categories.

#### 2.5.10 Sensitivity analysis

We will conduct a sensitivity analysis to identify whether the conclusions are robust in the review according to the following criteria: sample size, heterogeneity qualities, and statistical model (random-effects or fixed-effects model).

#### 2.5.11 Grading the quality of evidence

The Grade of Recommendations Assessment, Development and Evaluation (GRADE) will be a tool to evaluate the quality of the evidence,^[17]^ and will rate the quality by the following levels: very low, low, moderate, or high 4 levels.

## 3 Discussion

Gastric ulcers have a high incidence in humans.^[18]^ In many cases, antibiotics are not necessary. As a noninvasive external physiotherapy, acupuncture is widely used for gastric ulcers from ancient times to modern, and is popular in China because of its simplicity, convenience, low cost, and so on.^[19]^ In recent years, an increasing number of clinical reports have been published on the treatment of gastric ulcers, but high-quality trials are still insufficient.^[20,21]^ This review will begin when necessary trials are met. To provide compelling evidence and better guide in clinical practice, all actions of this review will be performed according to the Cochrane Handbook 5.2.0.

## Author contributions

HW and HJ had the original idea of this work and drafted the protocol. JZ and JC designed the search strategies. FW proposed some advice for design and revision. TL designed a flow chart. All authors critically revised the draft and approved the final manuscript.

**Conceptualization:** Heran Wang, Hailin Jiang.

**Data curation:** Jinying Zhao, Meng Meng.

**Formal analysis:** Heran Wang, Hailin Jiang, Jinying Zhao.

**Funding acquisition:** Hailin Jiang, Fuchun Wang, Xiaona Liu.

**Investigation:** Heran Wang, Hailin Jiang.

**Methodology:** Hailin Jiang, Jiapeng Chai.

**Project administration:** Fuchun Wang, Tie Li.

**Supervision:** Fuchun Wang.

**Validation:** Heran Wang, Ting Pan.

**Visualization:** Hailin Jiang, Kaiyang Xu.

**Writing – original draft:** Heran Wang, Hailin Jiang.

**Writing – review & editing:** Heran Wang, Fuchun Wang, Tie Li.
